# Identification among morphologically similar *Argyreia* (Convolvulaceae) based on leaf anatomy and phenetic analyses

**DOI:** 10.1186/s40529-017-0178-6

**Published:** 2017-06-02

**Authors:** Paweena Traiperm, Janene Chow, Possathorn Nopun, G. Staples, Sasivimon C. Swangpol

**Affiliations:** 1 Department of Plant Science, Faculty of Science, Mahidol University, Rama VI Road, Ratchathewi, Bangkok, 10400 Thailand; 2 Department of Pharmaceutical Botany, Faculty of Pharmacy, Mahidol University, Bangkok, 10400 Thailand; 3 HRA, Herbarium, Royal Botanic Gardens, Kew, Richmond, Surrey TW9 3AE UK

**Keywords:** Leaf epidermis, Leaf transverse section, PCA, SEM, Species complex, Stomatal index, UPGMA

## Abstract

**Background:**

The genus *Argyreia* Lour. is one of the species-rich Asian genera in the family Convolvulaceae. Several species complexes were recognized in which taxon delimitation was imprecise, especially when examining herbarium materials without fully developed open flowers. The main goal of this study is to investigate and describe leaf anatomy for some morphologically similar *Argyreia* using epidermal peeling, leaf and petiole transverse sections, and scanning electron microscopy. Phenetic analyses including cluster analysis and principal component analysis were used to investigate the similarity of these morpho-types.

**Results:**

Anatomical differences observed between the morpho-types include epidermal cell walls and the trichome types on the leaf epidermis. Additional differences in the leaf and petiole transverse sections include the epidermal cell shape of the adaxial leaf blade, the leaf margins, and the petiole transverse sectional outline. The phenogram from cluster analysis using the UPGMA method represented four groups with an *R* value of 0.87. Moreover, the important quantitative and qualitative leaf anatomical traits of the four groups were confirmed by the principal component analysis of the first two components. The results from phenetic analyses confirmed the anatomical differentiation between the morpho-types.

**Conclusions:**

Leaf anatomical features regarded as particularly informative for morpho-type differentiation can be used to supplement macro morphological identification.

## Background

The genus *Argyreia* Lour., one of the larger genera in Convolvulaceae with approximately 135 species, is mainly distributed in tropical Asia (Staples and Traiperm 2017). During preparation of a nomenclatural review of the genus, we found that some species of *Argyreia* are quite similar in their vegetative morphology leading to confusion in identification when only leaf and stem characters are available. Species delimitation in Convolvulaceae generally, including *Argyreia*, is heavily dependent on floral characters; when fully developed flowers are lacking it can be difficult or impossible to identify species precisely. Thus, when specimens with only leaves, or young flower buds, or fruits are all that is available it may not be possible to do more than narrow down the identification to a group of similar species. We have identified several such cases; these seem to represent species-complexes of morphologically similar species, or perhaps single highly polymorphic species. One such group of species includes *Argyreia breviscapa* (Kerr) Ooststr., *Argyreia kerrii* Craib, *Argyreia suddeeana* Traiperm & Staples, *Argyreia variabilis* Traiperm & Staples and two unnamed *Argyreia* that appear morphologically distinct. Previously we pointed out that some species complexes have similar macro morphological leaf characters (Staples and Traiperm 2008; Traiperm and Staples 2014). In order to explore other characters that might be useful for identification, this research investigates foliar micromorphology as a means to identify plants or specimens that lack flowers.

Leaf anatomy of some species in *Argyreia* had been investigated by Metcalfe and Chalk (1950), Sayeedud-Din (1953) and Pant and Bhatnagar (1975). Leaf epidermis and stem transverse sections of four *Argyreia* species [*Argyreia nervosa* (Burm.f.) Bojer, *Argyreia onilahiensis* Deroin, *Argyreia sericea* Dalzell & A. Gibson and *Argyreia splendens* (Hornem.) Sweet] were studied by Tayade and Patil (2003, 2012a). A key to species was constructed based on the epidermal characters such as cuticular striations, anticlinal cell walls, and basal cells of trichomes (Tayade and Patil 2003). Recent publication of a new species of *Argyreia* suggested that leaf epidermal characters, such as the pattern of cutin, can be used to distinguish *Argyreia albiflora* Staples & Traiperm from the morphologically similar species *Argyreia wallichii* Choisy (Staples et al. 2015). Anatomical characters strongly supported species identification in an investigation of *Merremia* section *Xanthips* (Pisuttimarn et al. 2013).

Anatomical information has been used for this analysis because it has proven useful and informative in assisting with taxonomic identification and classification in other plants (Thadeo et al. 2014). Leaf anatomical characters from several families also revealed similarities between plant species when using phenetic analysis [cluster analysis (CA) and principal component analysis (PCA)] (Nikolić and Mitić 1991; Breitwieser and Ward 1993; De Faria et al. 2010; Aghababaeyan et al. 2014; Moraes et al. 2011; Thadeo et al. 2014; Arthan et al. 2016; Jayarathna et al. 2016). In Convolvulaceae, particularly the genus *Argyreia*, phenetic analysis based on leaf anatomy has not been done before. The aim of this research was to study the leaf anatomy of *A. breviscapa* and similar-looking morpho-types and to conduct phenetic analyses using leaf anatomical characters to determine their utility for taxon discrimination and identification.

## Methods

### Plant collections

Plant materials were collected from their natural habitats for six morpho-types that are similar to *A. breviscapa* (e.g., *A. kerrii*, *A. suddeeana*, *A. variabilis*, *Argyreia* 1 and *Argyreia* 2). For each morpho-type, 1–5 populations were sampled. At least five mature leaves in each population were fixed in a mixture of 70% ethyl alcohol and 0.05% glycerol. Voucher specimens were prepared for each morpho-type and deposited at BKF (Table 1). Investigation of the micro-morphological characters was divided into three parts: epidermal surfaces examined by the peeling method; epidermal surfaces viewed with scanning electron microscopy (SEM); and leaf transverse sections prepared by modified paraffin method (Johansen 1940).

**Table 1:** List of the plant materials used in this study

No. of accession	Taxa	Voucher specimens	Locality
1	*Argyreia breviscapa* (Kerr) Ooststr.	J. Chow and P. Traiperm s.n.	Khon Kaen, Thailand
2		Staples et al. 1550	Kanchanaburi, Thailand
3	*A. kerrii* Craib	J. Chow and P. Traiperm 002	Chiang Mai, Thailand
4	*A. suddeeana* Staples & Traiperm	J. Chow and P. Traiperm 017	Ratchaburi, Thailand
5		J. Chow and P. Traiperm 019	Ratchaburi, Thailand
6	*A. variabilis* Traiperm & Staples	S. Suddee s.n.	Phetchaburi, Thailand
7		J. Chow and P. Traiperm s.n.	Phetchaburi, Thailand
8	*Argyreia* 1	J. Chow and P. Traiperm 030	Kanchanaburi, Thailand
9	*Argyreia* 2	J. Chow and P. Traiperm 031	Kanchanaburi, Thailand
10		J. Chow and P. Traiperm 032	Kanchanaburi, Thailand
11		J. Chow and P. Traiperm 033	Kanchanaburi, Thailand
12		J. Chow and P. Traiperm 034	Kanchanaburi, Thailand
13		J. Chow and P. Traiperm 035	Kanchanaburi, Thailand

### Leaf epidermal surfaces

Mature leaves were selected and peeled by a razor blade. The epidermis was stained with Safranin-O for 10‒15 min, and then dehydrated in an ethyl alcohol series, finally mounted with DePeX for a permanent slide. The stomatal index was calculated according to the method described by Salisbury (1927).

Leaf surfaces were also studied intensively by the scanning electron microscope (SEM). Small pieces of leaf (5 × 5 mm^2^) were dehydrated in ethanol series and sonicated to remove unwanted parts from the surfaces. After that, dried samples were coated with platinum-palladium in a sputter coater (Hitachi E-102 Ion Sputter). The cuticular patterns were observed and imaged under a Hitachi S-2500 scanning electron microscope.

### Leaf transverse section

The paraffin method used in this study was modified from Johansen (1940). Leaf samples were cut using a sliding microtome, stained with Safranin-O and counterstained with Fast Green. The tissues were dehydrated using ethanol series and mounted with DePeX.

The leaf characters from the epidermis and the transverse sections were observed using a light microscope, Olympus BX43, and photo-micrographed using an Olympus DP21 camera.

### Phenetic analyses

Thirteen accessions (Table 1) were used in the analysis. Twenty-four leaf anatomical characters (six qualitative and 18 quantitative) were analyzed from leaf surfaces, leaf and petiole transverse sections (Table 2). The qualitative traits were repeatedly observed under light microscopy with five different leaf sample slides per one accession to confirm character stability. For the quantitative characters, each character was measured and means with standard deviations were calculated in ten replicates. For reducing bias in quantitative characters, most of them were expressed in terms of ratios (except blade thickness).

**Table 2:** The leaf anatomical characters and character states used in phenetic analyses

Anatomical characters	Character states	Score
Qualitative characters		
1. Epidermal cell wall on adaxial leaf surface	Straight to slightly curved/sinuate	0/1
2. Epidermal cell wall on abaxial leaf surface	Straight to slightly curved/sinuate	0/1
3. Types of non-glandular trichome on abaxial leaf surface	Large and small straight trichome/large straight and small curly trichome	0/1
4. Shape of petiole	Slightly concave/convex	0/1
5. Midrib outline and adaxial curvature	Rounded/acute	0/1
6. Leaf margin shape	Rounded/acute	0/1
Quantitative characters		
7. Ratio of adaxial leaf epidermal cell size (width: length)	Ratio is less than or equal to 0.7/ratio is more than 0.7	0/1
8. Stomatal index (SI) on adaxial leaf surface	SI is less than or equal to 6/SI is more than 6	0/1
9. Ratio of stomatal size on adaxial leaf surface (width: length)	Ratio is less than or equal to 0.6/ratio is more than 0.6	0/1
10. Ratio of glandular trichome size on adaxial leaf surface (width: length)	Ratio is less than or equal to 0.92/ratio is more than 0.92	0/1
11. Ratio of non-glandular trichome size on adaxial leaf surface (width: length)	Ratio is less than or equal to 0.08/ratio is more than 0.08	0/1
12. Ratio of abaxial leaf epidermal cell size (width: length)	Ratio is less than or equal to 0.65/ratio is more than 0.65	0/1
13. Stomatal index (SI) on abaxial leaf surface	SI is less than or equal to 15/SI is more than 15, but less than or equal to 30/SI is more than 30	0/1/2
14. Ratio of stomatal size on abaxial leaf surface (width: length)	Ratio is less than or equal to 0.75/ratio is more than 0.75	0/1
15. Ratio of glandular trichome size on abaxial leaf surface (width: length)	Ratio is less than or equal to 0.9/ratio is more than 0.9	0/1
16. Ratio of non-glandular trichome size on abaxial leaf surface (width: length)	Ratio is less than or equal to 0.03/ratio is more than 0.03, but less than or equal to 0.04/ratio is more than 0.04	0/1/2
17. Ratio of epidermal cells size in petiole (width: length)	Ratio is less than or equal to 0.7/ratio is more than 0.7	0/1
18. Ratio of petiole transverse section (width: length)	Ratio is less than or equal to 0.82/ratio is more than 0.82, but less than or equal to 0.95/ratio is more than 0.95	0/1/2
19. Ratio of palisade mesophyll cell size in leaf transverse section (width: length)	Ratio is less than or equal to 0.2/ratio is more than 0.2	0/1
20. Ratio of spongy mesophyll cell size in leaf transverse section (width: length)	Ratio is less than or equal to 0.7/ratio is more than 0.7	0/1
21. Ratio of adaxial epidermal cell size in leaf transverse section (width: length)	Ratio is less than or equal to 0.7/ratio is more than 0.7	0/1
22. Ratio of abaxial epidermal cell size in leaf transverse section (width: length)	Ratio is less than or equal to 0.7/ratio is more than 0.7	0/1
23. Blade thickness (µm)	Thickness is less than or equal to 208/thickness is more than 208, but less than or equal to 248/thickness is more than 248	0/1/2
24. Ratio of midrib thickness (width: length)	Ratio is less than or equal to 1/ratio is more than 1	0/1

All data were transformed into discrete numbers to score and form the data matrix. The phenetic analyses including CA and PCA were performed. The cluster analysis was conducted based on Unweighted Pair Group Method with Arithmetic Mean (UPGMA) clustering in NTSYS-pc version 2.1 (Rohlf 2000). The similarity between morpho-types resulted in phenogram was estimated by simple matching coefficient (SM). The PCA was performed using Minitab version 16 (2010). The first and second components from PCA were presented for identification of the important anatomical traits.

## Results

### Epidermal cell wall and cuticle

**Fig. 1:**
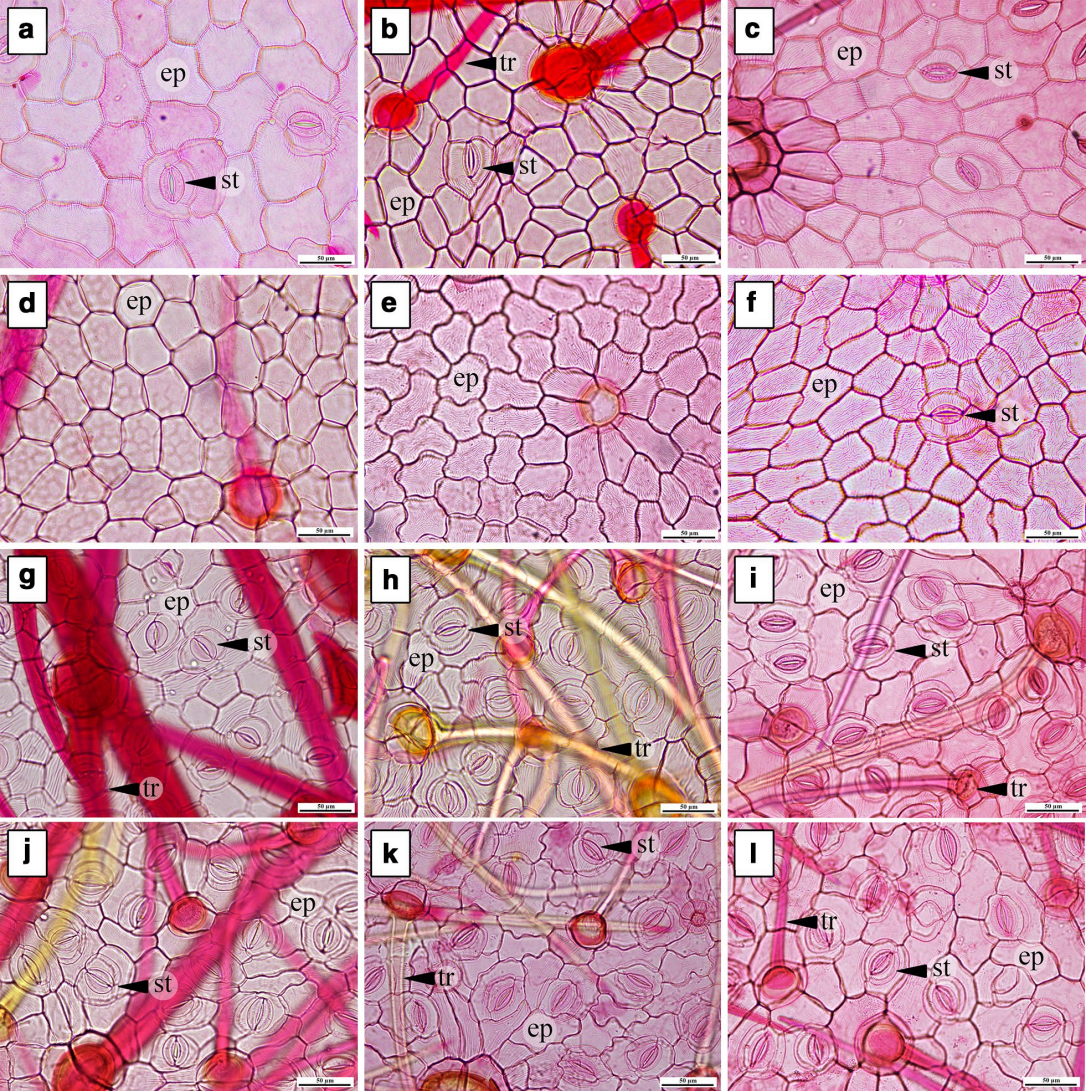
The epidermal cell walls on adaxial (**a**–**f**) and abaxial leaf surfaces (**g**–**l**) under light microscope (LM). **a** *A. breviscapa*, **b** *A. kerrii*, **c** *A. suddeeana*, **d** *A. variabilis* and **f** *Argyreia* 2, straight to slightly curved walls. **e** *Argyreia* 1, sinuate walls. **g** *A. breviscapa* and **j** *A. variabilis*, straight to slightly curved. **h** *A. kerrii*, **i** *A. suddeeana*, **k** *Argyreia* 1 and **l** *Argyreia* 2, sinuate (*ep* epidermal cell, *st* stomata, *tr* trichome). *Scale bar* 50 µm

**Fig. 2:**
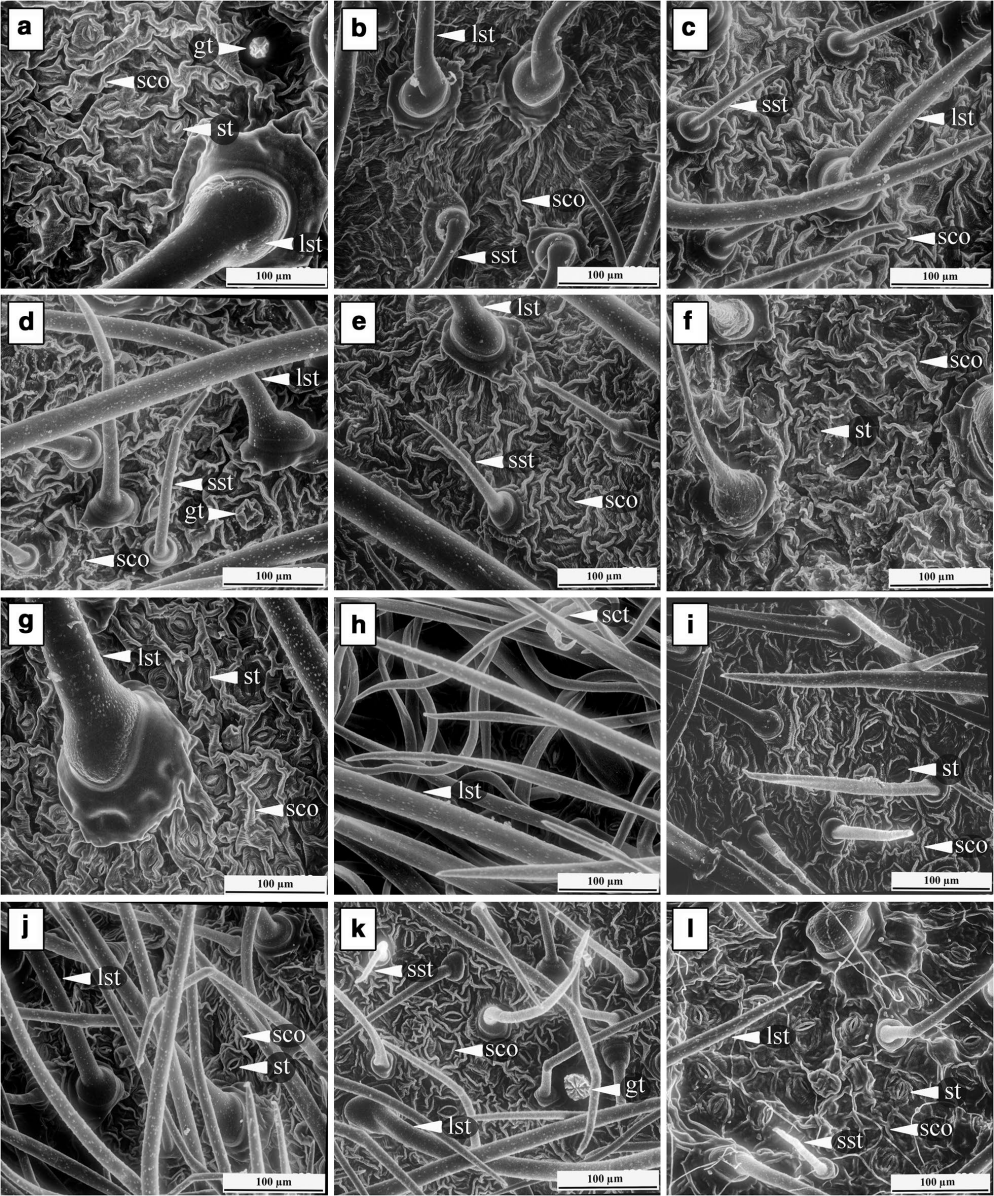
Cuticle ornamentation on adaxial (**a**–**f**) and abaxial leaf surfaces (**g**–**l**) from SEM. **a**, **g** *A. breviscapa*, **b**, **h** *A. kerrii*, **c**, **i** *A. suddeeana*, **d**, **j** *A. variabilis*, **e**, **k** *Argyreia* 1 and **f**, **l** *Argyreia* 2 (*gt* glandular trichome, *lst* large straight trichome, *sst* small straight trichome, *sct* small curly trichome, *st* stomata, *sco* striate cuticular ornamentation). *Scale bar* 100 µm

The patterns of anticlinal cell walls as seen under light microscopy appear straight to slightly curved and sinuate (Table 3). Almost all morpho-types have straight to slightly curved cell walls on adaxial surfaces, except in *Argyreia* 1, which shows a sinuate pattern of anticlinal cell walls (Fig. 1). Straight to slightly curved cell walls are present on the abaxial side of *A. breviscapa* and *A. variabilis* (Fig. 1g, j), whereas the others have sinuate patterns of anticlinal cell walls (Fig. 1h, i, k, l). The cuticular striation is shown on both sides of the lamina in all morpho-types (Fig. 2). However, *A. kerrii* possesses numerous trichomes on the abaxial leaf surface which interfere with viewing the surface (Fig. 2h).

**Table 3:** Leaf epidermal characters of all morpho-types in summary

Name	Adaxial epidermis						Abaxial epidermis					
	Stomatal index (S.I.)^a^	Epidermal cell wall	Cuticle	Stomatal type	Non glandular trichome and length (µm)	Density of trichome	Stomatal index (S.I.)^a^	Epidermal cell wall	Cuticle	Stomatal type	Non glandular trichome and length (µm)	Density of trichome
*A. breviscapa*	8.92 ± 2.76	Str-cur	Striated	Ani-para	LS = 546–820 SS = 311–440	+	24.92 ± 3.35	Str-cur	Striated	Ani-para	LS = 569–1291 SS = 249–487	++
*A. breviscapa*	13.68 ± 4.45	Str-cur	Striated	Ani-para	LS = 563–981 SS = 296–444	+	25.33 ± 6.87	Str-cur	Striated	Para	LS = 1053–1499 SS = 217–462	++
*A. kerrii*	2.42 ± 1.45	Str-cur	Striated	Para	LS = 512–738 SS = 280–402	++	22.87 ± 3.63	Sinuate	Striated	Para	LS and SC NT	+++
*A. suddeeana*	6.04 ± 2.23	Str-cur	Striated	Ani-para	LS = 515–945 SS = 128–410	+	26.84 ± 6.77	Sinuate	Striated	Para	LS = 583–1599 SS = 110–451	+++
*A. suddeeana*	7.19 ± 3.14	Str-cur	Striated	Ani-para	LS = 525–1037 SS = 149–313	+	25.70 ± 1.39	Sinuate	Striated	Para	LS = 667–1478 SS = 172–446	+++
*A. variabilis*	3.54 ± 1.05	Str-cur	Striated	Para	LS = 565–1100 SS = 155–478	+	33.21 ± 6.65	Str-cur	Striated	Para	LS = 796–1616 SS = 259–440	+++
*A. variabilis*	0.58 ± 1.32	Str-cur	Striated	Para	LS = 665–1295 SS = 145–420	+	25.46 ± 4.10	Str-cur	Striated	Para	LS = 948–1625 SS = 206–432	+++
*Argyreia* 1	4.37 ± 2.05	Sinuate	Striated	Ani-para	LS = 572–1042 SS = 142–391	+	22.22 ± 3.88	Sinuate	Striated	Para	LS = 617–1583 SS = 207–434	+++
*Argyreia* 2	5.21 ± 2.18	Str-cur	Striated	Ani-para	LS = 528–1073 SS = 197–428	+	27.68 ± 3.85	Sinuate	Striated	Para	LS = 708–1496 SS = 154–310	+++
*Argyreia* 2	10.71 ± 4.13	Str-cur	Striated	Ani-para	LS = 548–903 SS = 183–363	+	29.56 ± 3.08	Sinuate	Striated	Para	LS = 713–1673 SS = 173–305	+++
*Argyreia* 2	9.56 ± 1.32	Str-cur	Striated	Ani-para	LS = 551–1304 SS = 131–306	+	25.00 ± 2.77	Sinuate	Striated	Para	LS = 884–1994 SS = 144–424	+++
*Argyreia* 2	8.03 ± 2.47	Str-cur	Striated	Ani-para	LS = 544–824 SS = 173–390	+	24.42 ± 6.45	Sinuate	Striated	Para	LS = 685–1452 SS = 144–359	++
*Argyreia* 2	9.49 ± 4.75	Str-cur	Striated	Ani-para	LS = 523–945 SS = 160–318	+	25.00 ± 3.46	Sinuate	Striated	Para	LS = 661–1497 SS = 148–386	++

*NT* numerous trichome, *Str-cur* straight to slightly curved, *Ani-para* anisocytic and paracytic, *Para* paracytic, *10X* trichome density, + 1‒5 trichomes, ++ 6‒10 trichomes, +++ more than 10 trichomes, *LS* large straight trichome, *SS* small straight trichome, *SC* small curly trichome

^a^Mean ± SD

### Type of stomata and stomatal index

All of the morpho-types have amphistomatous leaves. The stomata on the adaxial leaf surface are anisocytic and paracytic in most of the morpho-types, excluding *A. kerrii* and *A. variabilis,* in which only the paracytic stomatal type was found. Conversely, a paracytic stomatal type presents on the abaxial leaf surface of every morpho-type, whereas *A. breviscapa* possesses both anisocytic and paracytic stomata (Table 3). On the adaxial epidermis the highest stomatal index was observed in *A. breviscapa* and the lowest in *A. variabilis*. On the other hand, the highest stomatal index on the abaxial epidermis was found in *A. variabilis* and the lowest in *Argyreia* 1 (Table 3).

### Type, density, size and length of trichomes

**Fig. 3:**
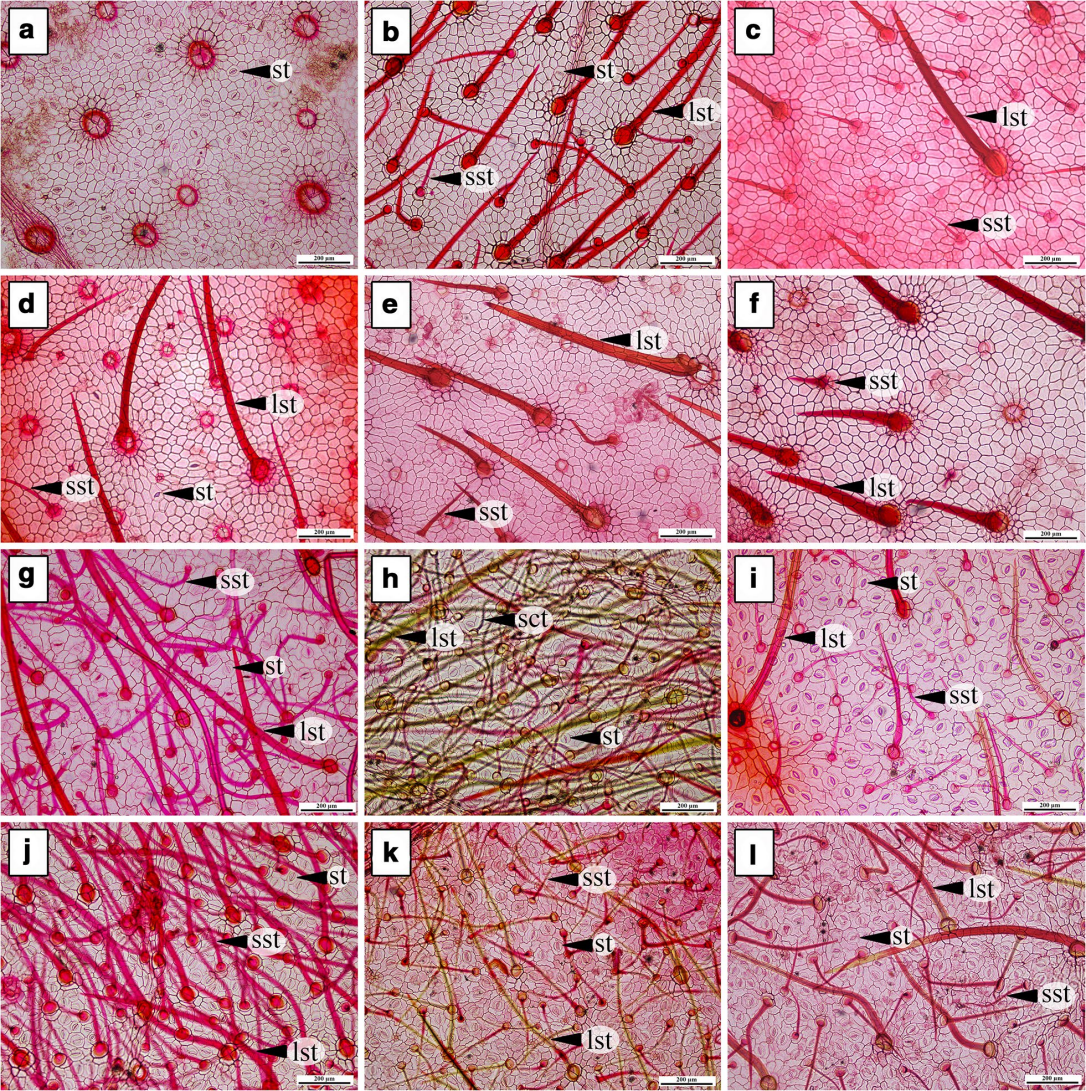
Trichomes on adaxial (**a**–**f**) and abaxial leaf surfaces (**g**–**l**) under light microscopy (LM). **a**, **g** *A. breviscapa*, **b**, **h** *A. kerrii*, **c**, **i** *A. suddeeana*, **d**, **j** *A. variabilis*, **e**, **k** *Argyreia* 1 and **f**, **l** *Argyreia* 2 (*lst* large straight trichome, *sst* small straight trichome, *sct* small curly trichome, *st* stomata). *Scale bar* 200 µm

Two types of trichomes are present in all morpho-types (Table 3): peltate glandular and simple non-glandular trichomes. Three types of non-glandular trichomes were recognized based on their length and direction: i.e. large straight; small straight; and small curly trichomes were found (Table 3). On their adaxial leaf surfaces, all of the morpho-types possess large and small, straight trichomes (Fig. 3a–f). Similarly on the abaxial leaf surfaces, most morpho-types have large and small straight trichomes excluding *A. kerrii,* which has large straight alternating with small curly trichomes (Fig. 3g–l). The longest trichomes on the adaxial epidermis occur in *A. variabilis*, while the shortest ones are in *A. suddeeana* (Table 3). Meanwhile, the longest trichomes on abaxial epidermis are in *A. breviscapa* and the shortest in *A. suddeeana* (Table 3).

### Transverse section of leaf blade and petiole

**Fig. 4:**
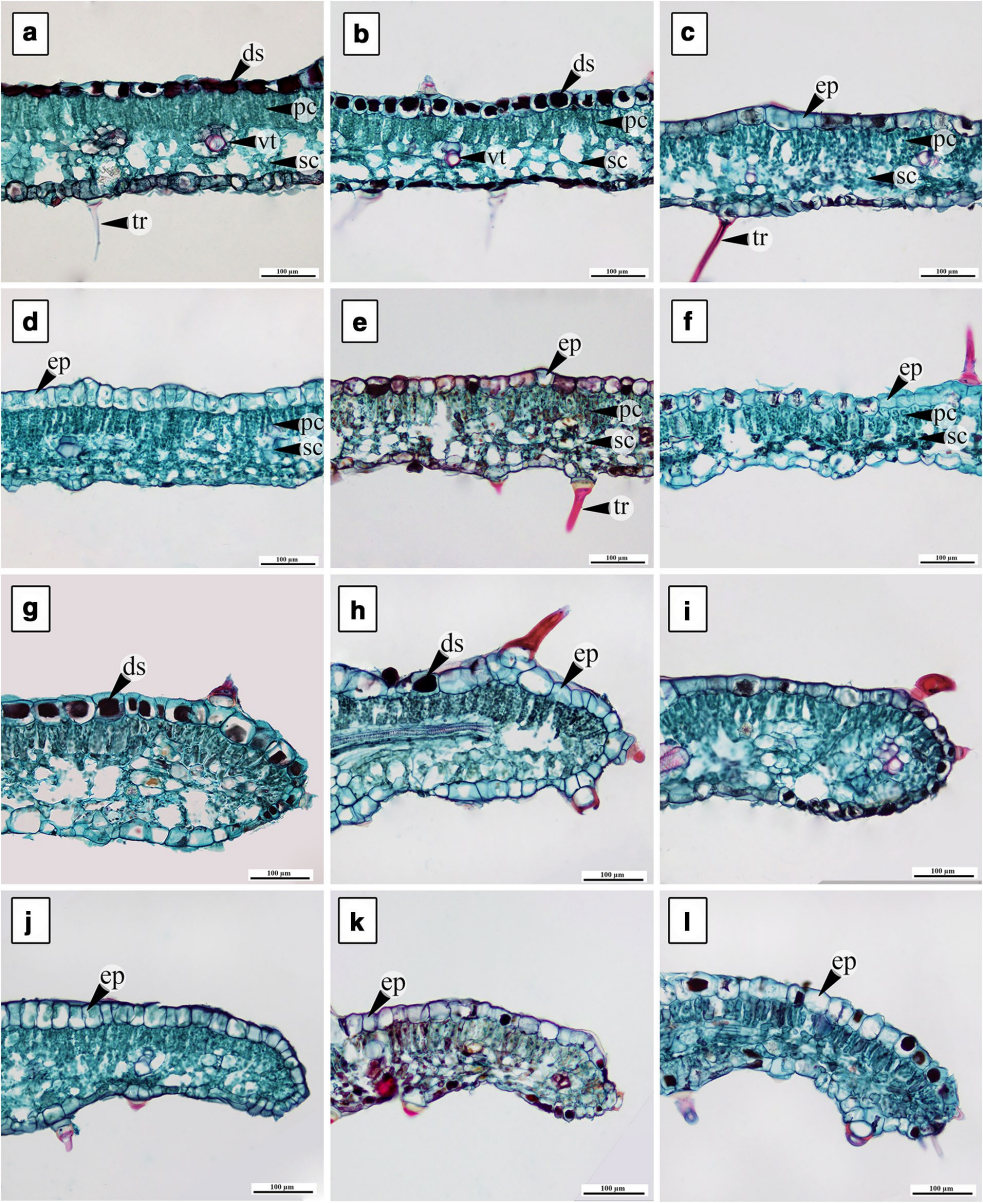
Transverse sections of leaf blade (**a**–**f**) and leaf margin (**g**–**l**). **a**, **g** *A. breviscapa*, **b**, **h** *A. kerrii*, **c**, **i** *A. suddeeana*, **d**, **j** *A. variabilis*, **e**, **k** *Argyreia* 1 and **f**, **l** *Argyreia* 2 (*ds* dark-staining deposit, *ep* epidermal cell, *pc* palisade cell, *sc* spongy cell, *tr* trichome, *vt* vascular tissue). *Scale bar* 100 µm

**Fig. 5:**
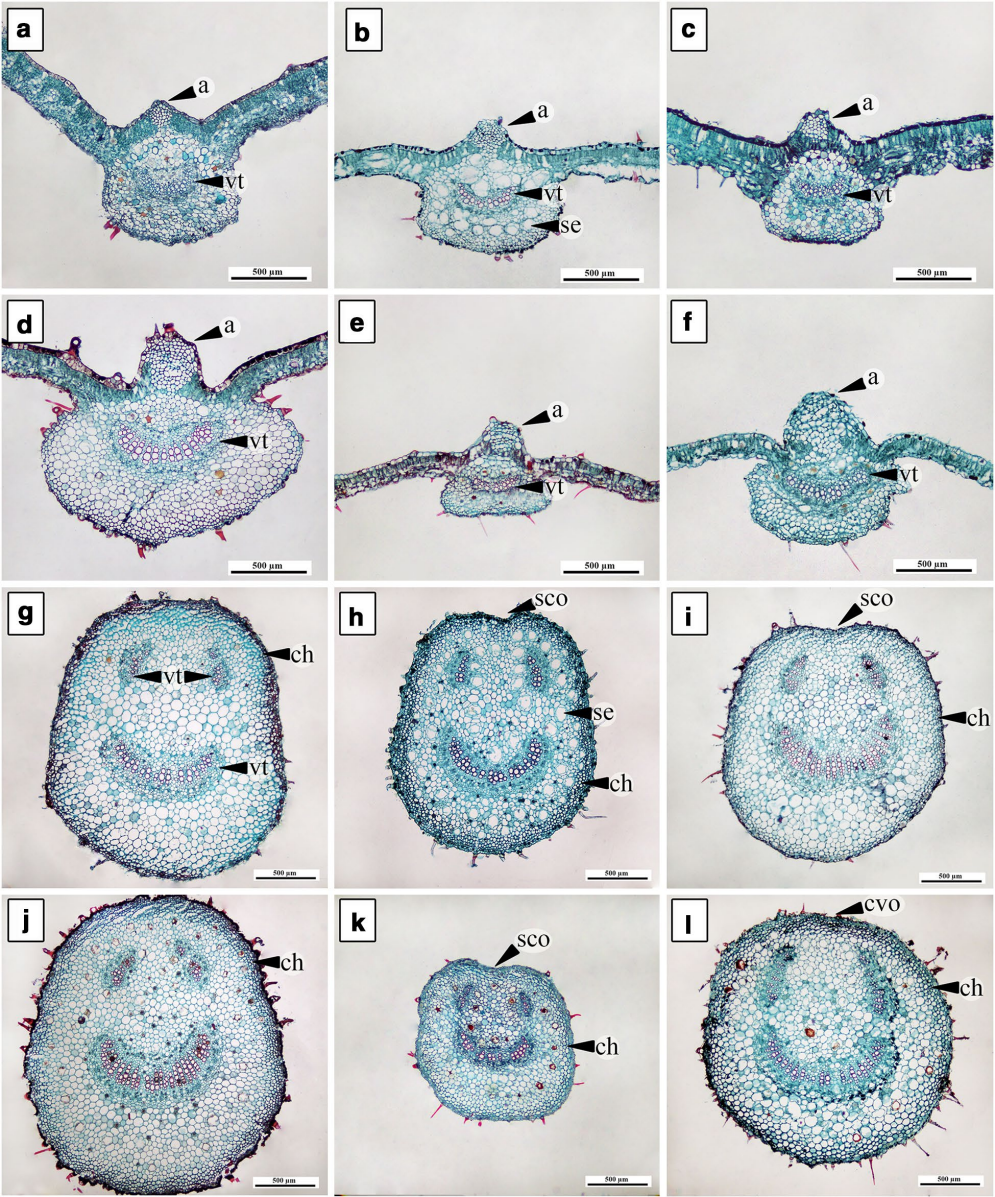
Transverse sections of midrib (**a**–**f**) and petiole (**g**–**l**). **a**, **g** *A. breviscapa*, **b**, **h** *A. kerrii*, **c**, **i** *A. suddeeana*, **d**, **j** *A. variabilis*, **e**, **k** *Argyreia* 1 and **f**, **l** *Argyreia* 2 (*a* adaxial curvature, *ch* chlorenchyma cell, *cvo* convex outline, *sco* slightly concave outline, *se* secretory cavity, *vt* vascular tissue). *Scale bar* 500 µm

Leaf blades of all six morpho-types have one layer of epidermis with thin cuticle (Fig. 4). Adaxial epidermal cells are irregular to round in shape excluding *A. variabilis*, which has long and narrow shaped cells (Fig. 4d). *Argyreia breviscapa*, *A. kerrii*, and *A. suddeeana* have dark-staining deposits in some of the cells on the adaxial surface (Fig. 5a–c). Palisade and spongy cells are different in shape. Palisade parenchyma is composed of 1‒2 cell layers. Most morpho-types have rounded leaf margins (Fig. 4g–i, k, l), except *A. variabilis,* which has acute margins (Fig. 4j) (Table 4).

**Table 4:** Leaf and petiole transverse section characters in summary

Name	Petiole outline	Cross-sectional petiole size (mm)		Midrib outline and adaxial curvature	Cross-sectional midrib size (mm)		Leaf blade width (µm)	Leaf margins
		Width	Height		Width	Height		
*A. breviscapa*	Convex	2.02 ± 0.05	2.10 ± 0.04	Convex with acute adaxial curvature	1.17 ± 0.24	1.03 ± 0.08	269.40 ± 35.64	Round
*A. breviscapa*	Convex	1.98 ± 0.02	2.28 ± 0.11	Convex with acute adaxial curvature	1.19 ± 0.08	1.14 ± 0.07	211.77 ± 7.84	Round
*A. kerrii*	Concave	1.73 ± 0.03	1.76 ± 0.23	Convex with round adaxial curvature	1.01 ± 0.06	0.95 ± 0.05	198.70 ± 45.96	Round
*A. suddeeana*	Concave	2.01 ± 0.17	2.06 ± 0.12	Convex with round adaxial curvature	1.40 ± 0.29	1.21 ± 0.15	183.03 ± 3.46	Round
*A. suddeeana*	Concave	1.51 ± 0.15	1.65 ± 0.12	Convex with round adaxial curvature	0.96 ± 0.31	0.92 ± 0.08	291.23 ± 28.90	Round
*A. variabilis*	Convex	2.24 ± 0.09	2.77 ± 0.39	Convex with round adaxial curvature	1.33 ± 0.12	1.34 ± 0.12	177.60 ± 29.23	Acute
*A. variabilis*	Convex	1.98 ± 0.58	2.34 ± 0.59	Convex with round adaxial curvature	1.52 ± 0.20	1.26 ± 0.15	244.87 ± 44.14	Acute
*Argyreia* 1	Concave	1.47 ± 0.18	1.24 ± 0.15	Convex with round adaxial curvature	0.85 ± 0.26	0.71 ± 0.10	162.53 ± 11.59	Round
*Argyreia* 2	Convex	1.99 ± 0.19	2.20 ± 0.02	Convex with round adaxial curvature	1.34 ± 0.17	1.26 ± 0.13	191.53 ± 9.76	Round
*Argyreia* 2	Convex	1.44 ± 0.09	1.82 ± 0.02	Convex with round adaxial curvature	0.91 ± 0.09	0.94 ± 0.06	171.83 ± 22.91	Round
*Argyreia* 2	Convex	1.52 ± 0.12	1.84 ± 0.04	Convex with round adaxial curvature	0.98 ± 0.12	1.05 ± 0.07	174.57 ± 33.96	Round
*Argyreia* 2	Convex	1.43 ± 0.09	1.83 ± 0.07	Convex with round adaxial curvature	0.91 ± 0.10	1.05 ± 0.01	156.73 ± 14.62	Round
*Argyreia* 2	Convex	1.73 ± 0.03	1.73 ± 0.23	Convex with round adaxial curvature	0.95 ± 0.09	0.92 ± 0.04	220.97 ± 16.15	Round

Outlines of the midrib are convex on the adaxial surface with a single acute to rounded adaxial curvature, while abaxial side are rounded or flattened. Almost all have rounded adaxial curvature (Fig. 5b–f), except *A. breviscapa* (Fig. 5a). Vascular tissue in the midrib is bicollateral type (Fig. 5a–f). Secretory cavities occur in all morpho-types, especially in *A. kerrii* (Fig. 5b). The largest cross-sectional midrib size is in *A. variabilis* and the smallest in *Argyreia* 1 (Table 4). Druse crystals are commonly found in the petioles, midribs, and leaf blades of all.

Petiole transverse section outlines are convex in *A. breviscapa*, *A. variabilis* and *Argyreia* 2 (Fig. 5g, j, l), while *A. kerrii*, *A. suddeeana* and *Argyreia* 1 show slightly concave outlines on the adaxial surface (Fig. 5h, i, k). Chlorenchyma comprises three to five cell layers in the cortex. Secretory cavities with thin walls are present in the ground parenchyma of all the morpho-types, particularly in *A. kerrii* (Fig. 5h). The vascular tissue of the petioles in all morpho-types consists of three bicollateral bundles. The largest cross-sectional petiole size is shown in *A. variabilis* and the smallest in *Argyreia* 1 (Table 4).

### Cluster analysis

**Fig. 6:**
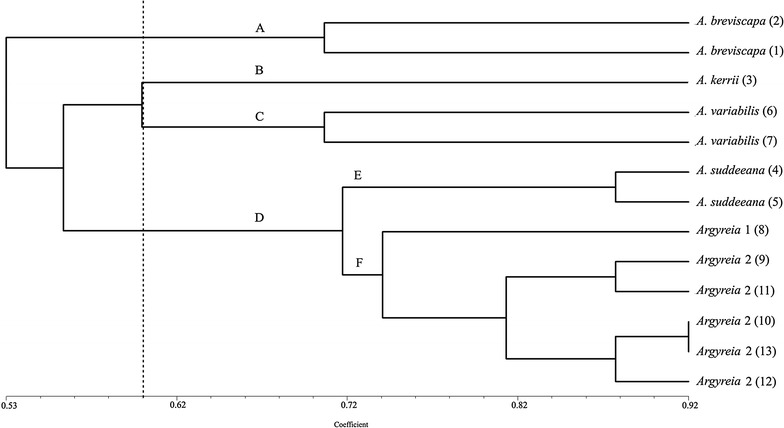
Phenogram for six *Argyreia* morpho-types from cluster analysis based on UPGMA method using 24 anatomical traits; the* vertical dashed line* indicates similarity coefficient; parenthetical numbers (*1*)–(*13*) indicate accession number of plant materials cited in Table 1

The phenogram of *Argyreia* morpho-types (Fig. 6) using 24 anatomical traits (Table 2) was constructed by UPGMA algorithm, for which the cophenetic correlation coefficient (or *R* value) was equal to 0.87. The high *R* value (>0.8) indicated the suitability of morpho-types clustering. From the phenogram, four major clusters (A, B, C, and D) were distinctly recognized by similarity coefficient from at least 0.60 and up to 0.70 (Fig. 6). The first cluster A comprises two accessions of *A. breviscapa*; it had a high similarity coefficient around 0.70. Cluster B contains only one morpho-type, *A. kerrii*, whereas the adjacent cluster C was separated from *A. kerrii* at similarity coefficient around 0.60. The cluster C contains two samples of *A. variabilis* that had a similarity coefficient about 0.71. The last cluster D was separated from the other clusters by a similarity coefficient about 0.72, and formed two subgroups. The subgroup E comprises two accessions of *A. suddeeana* that had a high similarity coefficient around 0.88. The second subgroup, F, is composed of two *Argyreia* morpho-types which are a single accession of *Argyreia* 1, and a group of five samples of *Argyreia* 2 that mutually diverged from each other with the similarity coefficient of 0.74. Five accessions of *Argyreia* 2 were obviously separated from *Argyreia* 1 at around 0.81 of similarity coefficient. Moreover, *Argyreia* 2 was subdivided into one subgroup (accession numbers 9 and 11) with high similarity coefficient of 0.88, and a second subgroup (accession numbers 10, 12 and 13) with the same value of similarity (0.88).

### Principal component analysis

**Fig. 7:**
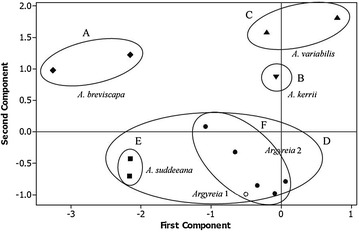
The scattered plot of first and second components obtained from principal component analysis (PCA) based on 24 anatomical traits. Group *A*, *B*, *C*, *D* and subgroups *E* and *F* corresponding to the phenogram in Fig. 6

The PCA resulting from examination of 24 anatomical characters (Table 5) from 13 *Argyreia* accessions representing six morpho-types showed the scatter plots of four clusters (Fig. 7). The two accessions of *A. breviscapa* in cluster A, and also both samples of *A. variabilis* in cluster C were quite loosely grouped, assuming that they were affected by the first component, while a single accession of *A. kerrii* was isolated from them. The large cluster, D, was formed into two subgroups, E and F. The morpho-types of subgroup E were closely grouped together, whereas those in subgroup F were widely separated by the first component.

**Table 5:** Factor loading and cumulative eigenvalue percentage received from 24 leaf anatomical characters

Anatomical characters	Component	
	1	2
1. Epidermal cell wall on adaxial leaf surface	0.024	−0.086
2. Epidermal cell wall on abaxial leaf surface	0.071	0.397
3. Types of non-glandular trichome on abaxial leaf surface	0.051	0.059
4. Shape of petiole	0.076	0.131
5. Midrib outline and adaxial curvature	−0.217	0.153
6. Leaf margin shape	0.146	0.245
7. Ratio of adaxial leaf epidermal cell size	0.051	0.059
8. Stomatal index (SI) on adaxial leaf surface	−0.193	0.067
9. Ratio of stomatal size on adaxial leaf surface	−0.124	0.281
10. Ratio of glandular trichome size on adaxial leaf surface	−0.202	0.118
11. Ratio of non-glandular trichome size on adaxial leaf surface	0.000	−0.163
12. Ratio of abaxial leaf epidermal cell size	0.093	0.172
13. Stomatal index (SI) on abaxial leaf surface	0.222	0.159
14. Ratio of stomatal size on abaxial leaf surface	0.043	0.113
15. Ratio of glandular trichome size on abaxial leaf surface	−0.197	−0.304
16. Ratio of non-glandular trichome size on abaxial leaf surface	−0.448	−0.229
17. Ratio of epidermal cell size in petiole	0.122	0.390
18. Ratio of petiole transverse section	−0.430	0.026
19. Ratio of palisade mesophyll cell size in leaf transverse section	0.076	0.063
20. Ratio of spongy mesophyll cell size in leaf transverse section	−0.114	0.284
21. Ratio of adaxial epidermal cell size in leaf transverse section	0.177	0.103
22. Ratio of abaxial epidermal cell size in leaf transverse section	0.108	−0.245
23. Blade thickness	−0.504	0.243
24. Ratio of midrib thickness	0.083	−0.156
Eigenvalue	1.3776	1.0784
Cumulative % of eigenvalue	28	49.9

The first two components accounted respectively for 28 and 21.9% of the total variance (Table 5). In the first component, the one character with high positive factor loading was stomatal index on the abaxial leaf surface (13), while high negative factor loading were the ratio of non-glandular trichome size on abaxial leaf surface (16); ratio of petiole transverse section (18); and blade thickness (23). The second component was weighted heavily positive for epidermal cell walls on abaxial leaf surface (2), and ratio of epidermal cell size in petiole (17), whereas it was negative for ratio of glandular trichome size on abaxial leaf surface (15), and ratio of non-glandular trichome size on abaxial leaf surface (16).

## Discussion and conclusions

The anatomical characters that are informative for identifying these six morpho-types are the epidermal cell walls and trichome types on the leaf surfaces. Moreover, the characters from leaf transverse section were also useful to distinguish each morpho-type such as the shape of adaxial epidermal cells of the leaf blades, the shape of leaf margins and the outline of petioles.

### Epidermal cell wall

Two types of epidermal cell walls are found on both surfaces: straight to slightly curved, and sinuate. This finding is in agreement with the investigations by Metcalfe and Chalk (1950) and Tayade and Patil (2003, 2012c). *Argyreia* 1 is the only one that possesses sinuate epidermal cell walls on the adaxial surface, whereas most of them have this type of epidermal cell wall on the abaxial side, except *A. breviscapa* and *A. variabilis*. *Argyreia breviscapa* is quite similar to *Argyreia* 1 in terms of morphological characters, especially when compared as pressed and dried herbarium specimen. This study shows that observation of the epidermal cell walls can separate *A. breviscapa* from *Argyreia* 1, and cluster analysis (CA) further revealed that these two morpho-types are placed in different clusters with similarity coefficient of 0.53 (Fig. 6). This suggests that the two morpho-types are not the same taxon. Further investigations, using different techniques such as molecular genetics, are required for more accurate characterization.

### Type of stomata and stomatal index

Plants in the family Convolvulaceae generally have amphistomatic leaves with paracytic type of stomata, rarely anisocytic (Metcalfe and Chalk 1950; Tayade and Patil 2003, 2012b). The results from this study agreed with the previous researches; nevertheless, anisocytic stomata were present in four morpho-types. The stomatal frequency on both leaf surfaces varies in each morpho-type; this is considered systematically significant and may be connected with the ecology of the plants (Stace 1965). Commonly, the stomatal index (SI) might vary depending on light intensity, humidity, and level of air pollution (Nikolić and Mitić 1991). However, both the stomatal frequency and stomatal index were treated as useful characters for diagnostic purposes (Metcalfe and Chalk 1979).

### Trichomes and cuticular pattern on leaf surfaces

Trichomes are known to play essential roles in protecting plants from chilling, drought, UV radiation, disease infection, and particularly against insect herbivory (Liakoura et al. 1997; Pfeiffer et al. 2003). Furthermore, trichome density may vary due to genetic as well as environmental factors, including water and mineral nutrients or the accumulation of water which alters leaf size (Johnson 1975; Wilkens et al. 1996; Roy et al. 1999).

Both glandular and non-glandular trichomes are present in *Argyreia*. Trichomes are normally present on both leaf surfaces in the six morpho-types studied. Finding glandular peltate trichomes in all morpho-types corresponded to reports in the previous studies by Tayade and Patil (2012a, c). Non-glandular trichomes, both large and small straight unicellular types, are found in all the morpho-types, but the large straight and small curly trichomes occur only on the abaxial surface of *A. kerrii*. The CA result also confirmed that *A. kerrii* is separated from cluster C with a 0.602 similarity coefficient (Fig. 6).

Cuticular striations occur in all six morpho-types studied. The diversity of patterning on the cuticular layers can be conveniently employed in taxonomic delineations in this family, as suggested by Tayade and Patil (2003). Our recent paper showed that the cutin layer can distinguish two morphologically similar species, *A. albiflora* and *A. wallichii* (Staples et al. 2015).

### Transverse sections of leaf blade and petiole

Characters obtained from leaf transverse sections in this research are in agreement with previous studies (Metcalfe and Chalk 1950; Tayade and Patil 2012b, c) that demonstrated all *Argyreia* leaves are dorsiventral; the leaf blades of every morpho-type have one layer of epidermis; and the mesophyll is composed of palisade and spongy cells. Additionally, there are some dark-staining deposits, which could be flavonoids or alkaloids, in some of the epidermal cells on the adaxial surface. This occurrence corresponded to the study of loline alkaloids in *Argyreia mollis* (Burm.f.) Choisy, which also can be found in roots and aerial vegetative parts of plants (Tofern et al. 1999; Mann et al. 1999; Tayade and Patil 2012b).

The epidermal cell shapes of leaf blade in transverse section of *A. variabilis* show long and narrow shapes and its leaf margins are acute, which is different from the other morpho-types. This coincides with phenetic result that show two accessions of *A. variabilis* separated from cluster B (Fig. 6) in cluster analysis by similarity coefficient of 0.602 and forming a distinct cluster C. The epidermal cell shape character and results from phenetic analyses can be used to point out that *Argyreia* 2 is different from *A. variabilis*.

The vascular tissue of the petiole in the family Convolvulaceae commonly exhibits bicollateral bundles (Metcalfe and Chalk 1950; Tayade and Patil 2012a, c). The results from this research correspond to the prior studies.

Shape of the *Argyreia* petiole in transverse section is convex (Fig. 5g, j, l) or slightly concave (Fig. 5h, i, k) on the adaxial side, as also seen in the study of genus *Merremia* Dennst. ex. Endl. (Pisuttimarn et al. 2013). In our results, half the taxa have convex petiole shape (*A. breviscapa*, *A. variabilis*, and *Argyreia* 2), whereas in *A. kerrii*, *A. suddeeana* and *Argyreia* 1, petioles have a concave shape (about 1/6 of the length of the petiole). The result from CA indicated that *A. suddeeana*, *Argyreia* 1, and *Argyreia* 2 were grouped together in the same cluster (D), however, *A. suddeeana* was separated from this group forming a subgroup E, at similarity coefficient about 0.718 (Fig. 6). Therefore, the outline of petiole can be used to separate *A. suddeeana* from *Argyreia* 2.

Druse crystals (calcium oxalate) are commonly found in petiole, midrib, and leaf blade of all six morpho-types, which agrees with the previous investigations by Metcalfe and Chalk (1950), Sayeedud-Din (1953) and Tayade and Patil (2012a, c). The druse crystal has many functions in plants including calcium regulation, ion balance, tissue support, plant protection, detoxification and light assembly and reflection (Franceschi and Horner 1980; Nakata 2003). Recent research about druse crystals in a C_4_ plant, *Amaranthus hybridus* L., revealed that the plant used calcium oxalate crystals as a non-atmospheric carbon source in photosynthesis assimilation during the day, especially in drought condition (Tooulakou et al. 2016). In the daytime, druse crystals were degraded and provided subsidiary carbon used in photosynthesis under ambient conditions that caused stomatal closure (for preventing water loss). Moreover, these crystals were recovered during the night as a biochemical carbon reservoir.

Numerous secretory cavities in the ground tissue of the petiole and midrib are also observed in *A. kerrii*, in agreement with previous researches, which reported that plants in this family possess secretory cavities in the ground tissue of leaf, petiole, and stem (Metcalfe and Chalk 1950; Sayeedud-Din 1953; Tayade and Patil 2012a, c).

Although the results from this study were consistent with earlier anatomical studies they incrementally increase the fundamental knowledge base about the genus *Argyreia* and point the way for further study. Rather few plant samples in genus *Argyreia* were examined previously; in this study, we investigated several more *Argyreia* taxa that have not been studied before. Furthermore, our data demonstrate that anatomical traits can provide informative characters that have a useful role for plant identification. *Argyreia* has proven to be an extraordinarily complex genus and species delimitation is difficult, especially in the complexes of vegetatively similar species where floral characters have been the only reliable method to discriminate taxa. Accordingly, evidence from methods and techniques outside the usual morphological ones must be explored to determine whether they afford taxonomically informative characters. Since the genus *Argyreia* is one of the larger genera in the family Convolvulaceae comprising around 135 species (Staples and Traiperm 2017) the results from our study expand the baseline for understanding morphological and anatomical diversity within and between species.
